# Oral Cancer and Precancer: A Narrative Review on the Relevance of Early Diagnosis

**DOI:** 10.3390/ijerph17249160

**Published:** 2020-12-08

**Authors:** Silvio Abati, Chiara Bramati, Stefano Bondi, Alessandra Lissoni, Matteo Trimarchi

**Affiliations:** 1 Dentistry and Stomatology-IRCCS San Raffaele Hospital, University Vita-Salute, 20132 Milano, Italy; lissoni.alessandra@hsr.it; 2 School of Medicine, University Vita-Salute, 20132 Milano, Italy; bramati.chiara@hsr.it (C.B.); trimarchi.matteo@hsr.it (M.T.); 3 Otorhinolaryngology-Head & Neck Surgery Department, San Raffaele Hospital, University Vita-Salute, 20132 Milano, Italy; bondi.stefano@hsr.it

**Keywords:** oral health, oral pathology, primary prevention, oral healthcare, risk factors, oral cancer, pre-malignancies

## Abstract

Oral cancer (OC) is an uncommon malignancy in Western countries, being one of the most common cancers in some high-risk areas of the world. It is a largely preventable cancer, since most of the different risk factors identified, such as tobacco use, alcohol consumption, and betel nut chewing, are behaviors that increase the likelihood of the disease. Given its high mortality, early diagnosis is of utmost importance. Prevention and the anticipation of diagnosis begin with identification of potentially malignant lesions of the oral mucosa and with local conditions promoting chronic inflammation. Therefore, every lesion must be recognized promptly and treated adequately. The clinical recognition and evaluation of oral mucosal lesions can detect up to 99% of oral cancers/premalignancies. As stated by the World Health Organization, any suspicious lesion that does not subside within two weeks from detection and removal of local causes of irritation must be biopsied. Surgical biopsy remains the gold standard for diagnosis of oral cancer. Adjunctive tools have been developed and studied to help clinicians in the diagnostic pathway, such as toluidine blue vital staining and autofluorescence imaging. In the near future other methods, i.e., identification of salivary markers of progression may help in reducing mortality due to oral cancer.

## 1. Introduction

Oral cancers are squamous cell carcinomas (OSCCs) in more than 90% of cases [1]; other tumors of the oral cavity include those of the salivary minor glands, melanomas, and lymphomas [2]. OSCC can have various levels of differentiation and often give rise to node metastases. Lymphatic spreading into the neck is directly related to the T stage as well as the depth of invasion and tumor thickness [3,4].

Oral cancers, along with oropharyngeal cancers, are the sixth most common malignancy worldwide [1]. Globally, over 400,000 estimated new cases of oral cancer are diagnosed each year, two-thirds of which occur in Asian countries, such as Sri Lanka, Indonesia, India, Pakistan, and Bangladesh [1,5]. In these high-risk countries, oral cancer is the most common malignancy, accounting for over 25% of all new cases of cancer each year [1]. The incidence of oral cancer increases with age and is highest over 60 years, even though cases in people younger than 40 years are increasing [1].

Oral cancer has poor prognosis, with overall 5-year survival rates as low as 40%, although, if diagnosed in the early stages (I and II), survival rates can exceed 80% [1,6]. Up to 50% of oral cancers are diagnosed at an advanced stage (stage III and IV), as most patients are not symptomatic in the early stages and do not seek medical help until they show clear symptoms such as pain, bleeding, or a mass in the mouth or neck if lymphatic spread is already present [7]. When the diagnostic delay exceeds one month, the risk of having an advanced stage oral cancer stage is significantly higher [8]. In most cases, the patient is responsible for a large part of the diagnostic delay; however, delay can also be the result of an incorrect medical approach by not suspecting an oral malignancy and not diagnosing and treating it promptly and adequately [8,9,10]. As a general rule, prognosis worsens as the disease becomes more advanced and as the site of the tumor becomes less accessible (i.e., lip cancer has better survival rates than oropharyngeal cancer) [11]. Clinical and pathological stage at diagnosis remains the most important factor influencing prognosis [11]. Given the high mortality rate, early detection of oral malignancy and anticipation of diagnosis results in better prognosis and survival rates and less morbidity from treatment [12].

Risk factors for oral cancer typically include heavy use of tobacco (including smokeless tobacco), betel quid chewing, consumption of alcoholic beverages, and chronic inflammation [13,14,15,16,17]; the prevalence of HPV-associated (mainly HPV type 16) oral and oropharyngeal cancer has been increasing in recent decades, predominantly among younger people [11,18,19]. The oncogenic role of oral microbiome, mucosal inflammation, and oral mucosal trauma from teeth and prosthetic devices have received increasing attention in several clinical and scientific studies [20]. For lip cancer, actinic ultraviolet radiation (UV), mainly UV-B, also plays a role [3]. Furthermore, genetic conditions like xeroderma pigmentosum, Fanconi’s anemia and ataxia-telangiectasia show increased risk, due to a deficit in DNA repair mechanisms [21].

Given that most risk factors can be eliminated, oral cancer can be considered as a largely preventable disease. However, its occurrence in patients not belonging to risk categories is still possible [19]. Primary prevention of oral cancer therefore consists in education of people on the limitation of behavioral risk factors, in discouraging tobacco usage and addiction and limitation of alcohol intake [1]. HPV vaccination can also be of importance, even though its effectiveness in not as well defined as it is in the prevention of anogenital and cervical cancer [11]. When the carcinogenic process has already started, the mainstay of secondary prevention consists in screening and early treatment of oral premalignancies and early-stage cancers [1]. Despite the increasing awareness of oral cancer in the general population, in the last 40 years the percentage of patients seeking medical attention with advanced disease has not changed significantly [9].

Unlike other frequent cancers (i.e., colorectal or cervical cancer), a standardized population-based screening program for oral cancer is not cost-effective and cannot be recommended [22]. Screening programs in patients belonging to high-risk groups (heavy smokers and drinkers) or in patients with previous diagnosis of cancer outside the head and neck area could however be worthwhile [23], as shown by a randomized controlled trial in India [24]. In countries with regular dental practice attendance, opportunistic screening for oral mucosal lesions (early-stage cancer or precancerous lesions) in general dental practice could also be relevant in reducing diagnostic delay [25].

## 2. Potentially Malignant Lesions of the Oral Mucosa

Potentially malignant oral epithelial lesions (PMOELs) are a group of oral conditions and diseases that can be present before the onset of OSCC and include a group of clinically suspect oral mucosal lesions such as leukoplakia, erythroplakia, submucosal fibrosis, and lichen planus. The majority of PMOELs, however, do not progress to cancer [26].

### 2.1. Leukoplakia

In 1978, the WHO defined oral leukoplakia as a white patch of the oral mucosa that cannot be rubbed off and classified otherwise, either clinically or histopathologically [27]. Leukoplakia does not refer to the presence or absence of any stage of epithelial dysplasia, as it is a clinical definition and is not linked to a specific histology. Thus, it is considered as a diagnosis of exclusion, as any other characterization defines the lesion as something else [21,27,28,29]. White patches of the oral mucosa for which it is possible to identify a cause must be therefore excluded [30], such as lesions associated with local trauma, candidiasis, lichen planus, etc. [31]. The prevalence of leukoplakia has been reported to be 1–3%, with a higher incidence in men and a peak of occurrence in the fifth to seventh decade [32]. The most common locations in which these lesions usually appear are the alveolar mucosa, followed by the buccal mucosa, palate, tongue, and floor of the mouth [32]. It is not uncommon for a patient to have multiple lesions [32].

Leukoplakia is essentially an idiopathic condition [30], although, as for OSCC, tobacco smoking has been identified to be among the main risk factors for its occurrence; at the same time, this type of lesion can also occur in non-smokers. In cases in which smoking is linked to the occurrence of leukoplakia, cessation of smoking can cause the partial or complete regression of the lesion [31].

The transformation rate for leukoplakia is overall about 2–3% per year [31]; in patients presenting with these lesions, malignant transformation can also occur in other areas of the oral cavity and in the upper aerodigestive tract, and not only in the site of the lesion [31]. Leukoplakia is not only a premalignancy, but also a marker of a greater risk of cancer of the oral mucosa [32].

Oral leukoplakias can be classified clinically into two groups: homogeneous and non-homogeneous lesions [28,33]. Homogeneous leukoplakias are more common and mostly benign; they are uniform and flat in appearance, with small superficial cracks [28,33]. Non-homogeneous leukoplakias can have a range of different characteristics; they can either be flat and speckled, white and red in color (erythroleukoplakia), nodular, exophytic, or papillary/verrucous [28,33]. The distinction between the two types is purely clinical and is of utmost importance since it is known that non-homogeneous leukoplakias carry a much higher risk of malignant transformation [28]. Some authors consider the transformation rate of non-homogeneous lesions similar to that of erythroplakia [34]. Histopathology classifies most leukoplakias as benign keratosis, hyperkeratosis, or hyperplasia; only a minority show some degree of dysplasia or carcinoma [32]. As a general rule, the thicker the lesion, the greater the possibility of finding dysplasia in the sample [28,33].

**Figure 1:**
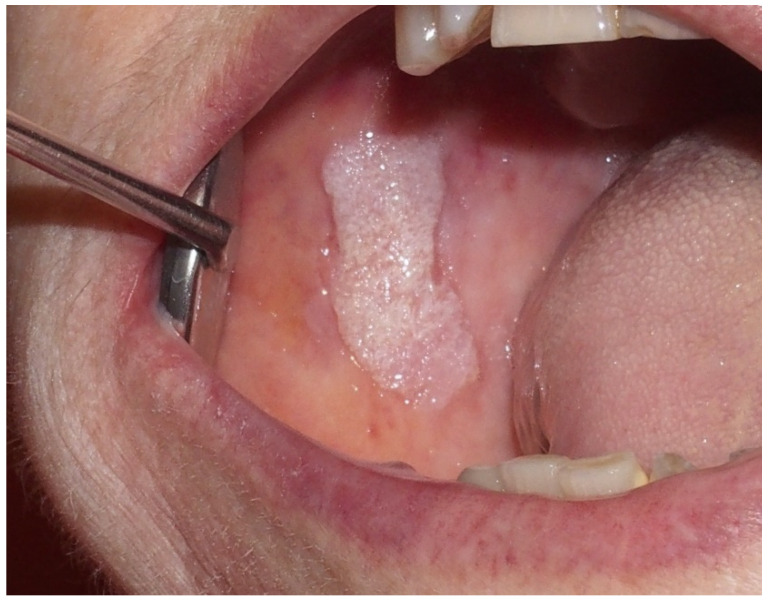
Female, 72 years—verrucous leukoplakia in the right buccal surface.

Proliferative verrucous leukoplakia (PVL) is a rare, high-risk, multifocal type of leukoplakia [35,36] mostly affecting women in their sixth decade of life with no history of tobacco or alcohol use [35,36]. PVL presents as non-homogeneous small white patches or plaques that evolve to become progressively verrucous end exophytic; the areas most commonly involved are the gingiva, alveolar mucosa, buccal mucosa, and tongue [35,36] (Figure 1). The malignant transformation rate has been reported to be 60% to 100% [35,36], with a higher rate of recurrence after surgical excision [37]. Due to its high chance of malignant transformation, it is extremely important to promptly recognize it through specific diagnostic criteria that include the following [35,38,39]:

1. Leukoplasic lesion present in more than 2 oral sites, most frequently the gingiva, alveolar processes, and palate;
2. Presence of a verrucous area;
3. Lesions that have spread or enlarged during development of the disease;
4. Recurrence in a previously treated area;
5. Exclusion of invasive OSCC with biopsy.

### 2.2. Erythroplakia

**Figure 2:**
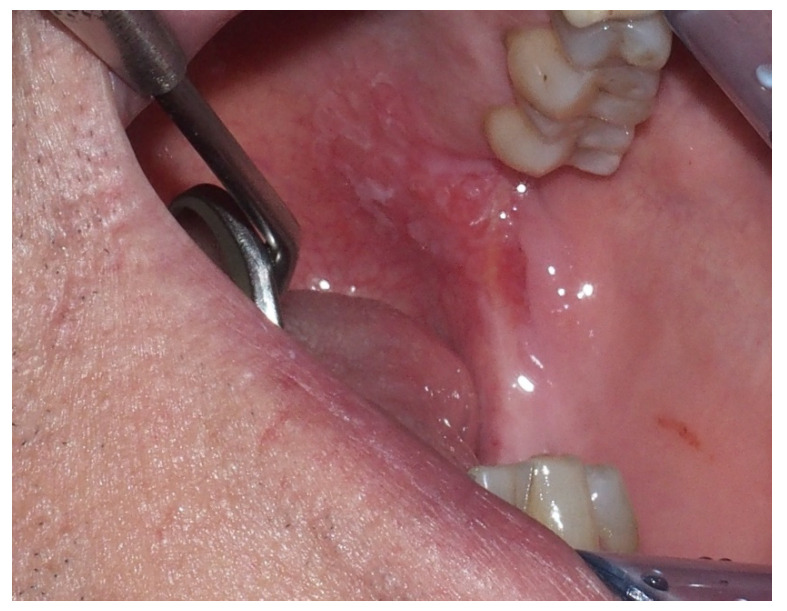
Male, 61 years—erythroplasic area in the posterior hard palate, revealed severe dysplasia and in-situ carcinoma in the histopathologic examination.

Erythroplakia presents as a red lesion usually well defined with a velvety texture, and much less frequent [7,40]; sometimes the lesion can have red and white areas, defined as erythroleukoplakia, speckled erythroplakia or leukoerythroplakia [7,26]. Erythroplakia enters into differential diagnosis with candidiasis, lichen planus, mucositis, and systemic lupus erythematosus [26]. Erythroplakic lesions are usually asymptomatic, but sometimes patients, most commonly those presenting with erythroleukoplakia [35], refer a burning sensation and/or soreness [26]. The most common site for the occurrence of this lesion is the soft palate, followed by the ventral tongue, floor of the mouth, and tonsillar pillars [36]; most patients present a single lesion [35] (Figure 2).

Erythroplakia is not as common as leukoplakia; its prevalence in the general population is not well known and has been reported to be between 0.02% and 0.2% [26]. Risk factors include those of oral cancer, such as tobacco smoking and alcohol intake [26]. The transformation rate of erythroplakia is not completely known, but it is thought to be much higher than leukoplakia [21], as up to 85% of erythroplakias show histological signs of malignancy—including carcinoma in situ and invasive carcinoma—at the time of biopsy [33]; for this reason, some authors claim that any lesion of the oral mucosa appearing red and velvety, with or without white components, should be considered as cancer—carcinoma in situ at the very least—until proven otherwise [41]. Given the high risk that erythroplakia carries, it is recommended to treat the condition promptly: either excisional or incisional biopsy is recommended, followed by complete removal of lesions showing severe dysplasia on histopathologic examination [36]. After excision, recurrence is possible, especially in larger lesions [36].

### 2.3. Oral Lichen Planus

**Figure 3:**
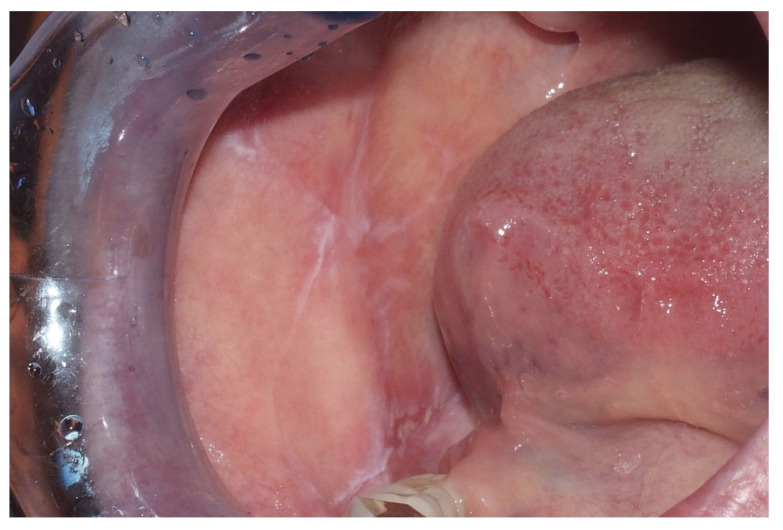
Male, 52 years—white reticular striae on the right cheek mucosa in a patient with oral lichen planus histopathologically confirmed.

Lichen planus is a chronic inflammatory disease immunologically driven that usually affects middle-aged patients [42], particularly women aged 30 to 60 [36]. It usually affects the skin, but can also have mucosal involvement, including the oral mucosa (oral lichen planus—OLP) [42]. Oral lichen planus is mostly a chronic disease, whereas the cutaneous form may remit within 6 to 12 months [36]. OLP can manifest in different ways and the subtypes can be classified as reticular (the most common type, characterized by Wickham striae and hyperkeratotic plaques or papules), papular, plaque, atrophic, erosive with areas of ulceration associated with keratotic white striae, and bullous, with bullae that can rupture and lead to ulceration [36,42]. The plaque subtype of oral lichen planus can resemble leukoplakia in appearance, which underlines the importance of biopsy [42]. OLP most commonly affects the buccal mucosa, followed by the gingiva and tongue; patients usually show multiple, bilateral lesions [36]. Erosive and atrophic OLP can cause severe pain and interfere with speech and swallowing [36] (Figure 3). It is still debated whether oral lichen planus can transform into oral squamous cell carcinoma [26], with reported transformation rates ranging between 0% and 12.5%: There are no clinical or histopathological characteristics that can help predict possible malignant transformation [42]. The erosive OLP carries the highest risk of malignant transformation, followed by atrophic OLP, while reticular OLP carries the lowest risk [36]. As for leukoplakia, cancers can arise in different areas of the oral cavity, and not strictly associated to the OLP lesions [21].

### 2.4. Submucosal Fibrosis

Submucosal fibrosis is a chronic fibrotic lesion of the oral mucosa. It is probably the expression of an overhealing wound, responding to chronic insults to the mucosal lining, either mechanical or chemical. This lesion is widely accepted to have malignant potential [28,43], with an estimated transformation rate of 9% [36]. Submucosal fibrosis is characterized by the loss of fibroelasticity in the affected tissue, resulting in palpable fibrous bands that can affect the mobility of the tongue and limit mouth opening [36]. This condition most commonly affects the buccal mucosa, followed by tongue, lip, palate, and gingiva; a predominance in males has been found [36]. Submucosal fibrosis was initially thought to be idiopathic but is now believed to have a multifactorial etiology, including capsaicin contained in chilies as well as iron, zinc, and vitamin deficiencies [44,45]. In India and east Asian countries, betel quid chewing has been found to be the main etiological factor [44], as the areca nut contains arecoline, a substance that stimulates fibroblasts, leading to fibrosis in the lamina propria [36]. As the disease advances, the fibrosis involves the deeper tissues of the submucosa, resulting in loss of fibroelasticity [39].

### 2.5. Chronic Inflammation

Chronic mucosal irritation has been postulated as an etiological factor for oral cancer. The associated chronic inflammation causes the release of mediators such as cytokines, leading to oxidative stress and subsequent damage to cellular DNA, resulting in a carcinogenic process [21].

Chronic trauma to the oral mucosa includes that due to broken and/or sharp teeth, ill-fitting dentures, prostheses, implants, and parafunctional habits (i.e., oral mucosa sucking, tongue thrusting) [46]. The areas of oral mucosa in contact with teeth or dental implants are those usually affected, with the lateral border of the tongue being the most common site for malignancies related to chronic inflammation [21].

**Figure 4:**
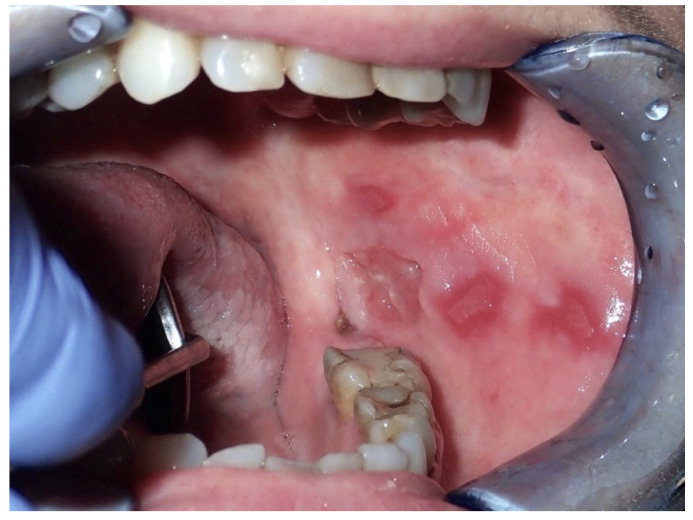
Female, 53 years—multiple areas of erythroplakia with ulceration in the left buccal mucosa; diagnosis of invasive squamous cell carcinoma, originated in a chronic traumatic ulcer from self-biting.

Chronic mucosal trauma has been recognized both as an initiative factor and as a progressor for oral cancer, as it can cause lesions on healthy mucosa or worsen existing lesions [21,46]. (Figure 4).

### 2.6. Oral Bacteria and Cancer

It is well established that oral hygiene is often particularly defective in patients affected by oral cancer [47].

Recent evidence shows that several associations exist between oral bacteria, mainly from periopathogenic biofilms, and oral cancerogenesis. Biological interactions between activities of bacteria from oral biofilms, i.e., *Fusobacterium nucleatum* and *Porphyromonas gingivalis* and epithelial cells are involved in this association. The mechanism proposed to explain the oncogenic potential are interference of bacteria with production and utilization of E-cadherin and adhesins, subversion of immunodefensive mechanisms, the effect of microorganisms on the host matrix metalloproteinases, gingipains molecules, and extracellular signal-regulated kinases, along with the suppression of apoptosis and stimulation of proliferation [47,48,49].

### 2.7. Other Potentially Premalignant Lesions

Other possible premalignant lesions of the oral mucosa include discoid lupus erythematosus, actinic keratosis (limited to lip cancer), hereditary conditions such as dyskeratosis congenita and epidermolysis bullosa, and chronic Candida infections [19,29].

### 2.8. Risk of Transformation

No PMOEL is a mandatory precursor to OSCC and most PMOELs do not transform into malignancies. It is not easy to discern PMOELs carrying a higher risk of transformation from those carrying a lower risk: the presence of dysplasia is considered to be the most useful indicator of possible malignant transformation [50,51]. However, it is important to keep in mind that non-dysplastic lesions may also transform into malignancies, while not all dysplastic lesions may progress to cancer [50].

**Figure 5:**
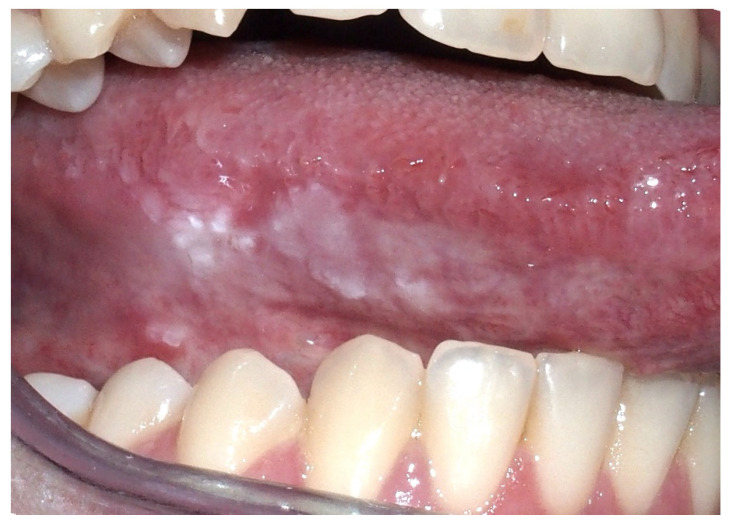
Female, 25 years—area of leukoplakia in the right border of the tongue with histologically confirmed severe dysplasia.

The presence or absence of dysplasia is not directly associated with a specific clinical appearance of the lesion, so it is not possible to predict whether dysplastic alterations are present before performing biopsy [51] (Figure 5).

Dysplasia can be defined as an alteration of the specific architectural characteristics of the mucosa and can be classified as mild, moderate, or severe, depending on the depth and severity of the alterations [50].

The changes in mucosa are related to DNA mutations affecting several different genes regulating cell signaling, growth, survival, motility, angiogenesis, and cell cycle control, leading to changes in cell growth [52]. This results in the progressive acquisition of a malignant phenotype that can lead to cancer [52]. Histologically, criteria for the diagnosis of dysplasia include irregular epithelial stratification, loss of polarity of basal cells, drop-shaped ridges, increased number of mitotic figures, abnormally superficial mitoses, dyskeratosis, and keratin pearls within rete pegs [53]. In mild dysplasia, these alterations are limited to the lower third of the epithelium, in moderate dysplasia they extend into the middle third, and in severe dysplasia, more than two-thirds of the epithelium is involved [53]. When dysplasic changes are severe and extend to the entire thickness and strata of the epithelium, a so-called carcinoma-in-situ, i.e., intraepithelial carcinoma is evident [51].

## 3. Early Diagnosis

Early detection and treatment of PMOELs, is of utmost importance in improving survival and reducing mortality [54]. The diagnostic process begins from clinical oral examination, which consists of visual inspection and digital palpation of the oral cavity [12,55]. A thorough clinical inspection of the oral cavity can detect up to 99% of oral cancers [54] (Table 1).

Suspect lesions must be further assessed: as initially proposed by the World Health Organization and the National Institute of Dental and Craniofacial Research and as stated in the recent evidence based protocol of the American Dental Association, any mucosal lesion persisting for two weeks or more, after removal of possible local irritants (broken teeth, ill-fitting dental prosthetic devices and appliances, dental plaque, etc.), must be biopsied, as histological examination is the gold standard in diagnosis of OSCC [27].

At present the routine cytologic examination of a smear collected from the epithelial surface of the oral mucosa does not have sufficient sensitivity and specificity to serve as a predictive diagnostic tool for squamous cell carcinoma, despite the low invasiveness of the sample collection. In the recent decades, more modern methods in oral cytology like brush-biopsy and microbiopsy have been proposed, which are useful mainly in the follow-up of precancerous lesions, avoiding the repetition of more invasive surgical biopsies [56].

Several other diagnostic adjunctive aids have been developed to help overcome the limits of standard oral clinical examination. While not as informative as biopsy, these methods can aid in suspecting and distinguishing between benign and potentially malignant lesions [54].

These testing methods may include toluidine blue staining, light-based detection techniques, and salivary biomarkers assessed with point of care devices.

Biopsy remains the gold standard, but could have a series of disadvantages, which discourage patients from agreeing to the procedure, including fear, stress, pain, and possible damage to healthy tissues, risk of infection, discomfort, temporary disability, and esthetic concerns [57].

**Figure 6:**
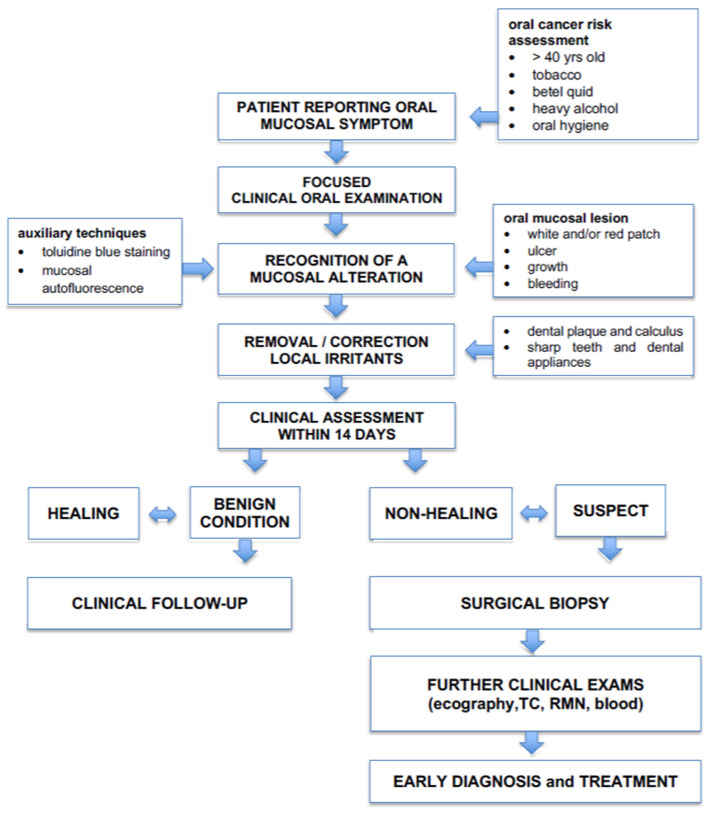
Clinical flow-chart to guide the clinician in anticipating the diagnosis of oral cancer and other mucosal diseases and conditions.

It is important to underline that clinical objective examination and surgical biopsy remain the most important and most effective diagnostic methods for oral cancer and pre-malignant lesions [54] (Figure 6).

### 3.1. Toluidine Blue Staining

Toluidine blue staining is a simple, inexpensive, and non-invasive technique used as an aid in the diagnosis of malignant and pre-malignant lesions of the oral cavity [58]. Toluidine blue is a cationic metachromatic dye that stains areas of the dysplastic epithelium, making them royal blue [57].

**Figure 7:**
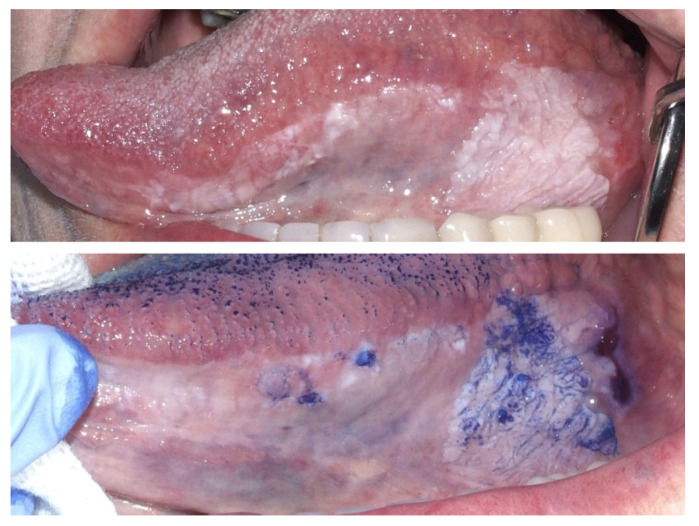
Male, 72 years—wide corrugated leukoplakia of the left border of the tongue; the application of toluidine blue staining revealed several foci of suspect epithelial dysplasia or cancer.

Its application is very easy and fast: a 1% aqueous solution is applied for 30 s on the area of the suspect lesion, after the application of 1% acetic acid to remove salivary and bacterial pellicle; the staining pattern is then evaluated [57] (Figure 7).

The mechanism by which toluidine blue binds to high-risk and malignant cells is not completely understood; some hypotheses include its affinity to nucleic acids, which results in its binding to cells containing high quantities of DNA and RNA, and its tendency to bind sulfated mucopolysaccharides [57,59]. Its sensitivity and specificity are, however, still debated, and are estimated to be between 72.5–84% and 61.4–70%, respectively, with one of its main drawbacks being its operator dependence [60]. Combining the results from toluidine blue staining with the information from clinical examination can increase the sensitivity to100% [59], especially for malignant lesions, while its sensitivity for pre-malignant lesions remains lower [57]. Its specificity, however, remains low, because ulcerations of the mucosa of any nature (traumatic, inflammatory, pre-neoplastic) tend to bind toluidine blue [59]. Toluidine blue positivity has also been found to be linked to worse prognosis, as lesions showing positivity tend to grow larger and result in cancer more often than negative lesions [61].

### 3.2. Autofluorescence Imaging

While clinical examination using white light remains one of the main tools in diagnosis of oral cancer, autofluorescence imaging may give additional information about the nature of the lesion, contributing to diagnosis and helping to discern lesions that warrant biopsy [62]. Several devices have been marketed in the last decades. They must be considered as adjuncts to visual and tactile clinical examination and cannot be considered replacements for it [63].

**Figure 8:**
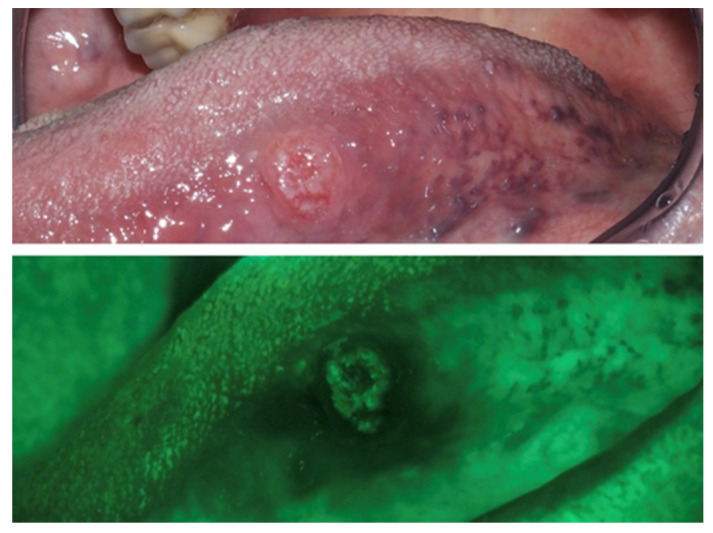
Male, 80 years—Histologically confirmed T1 squamous carcinoma of the left border of the tongue, with loss of autofluorescence if inspected with use of a fluorescence imaging device.

When excited with light of specific wavelengths, tissues produce autofluorescence, thanks to various endogenous fluorophores, including collagen, elastin, keratin, adenine dinucleotide (FAD), and nicotinamide adenine dinucleotide (NADH) [64]. The rationale for the use of autofluorescence imaging is the concept that dysplasia and cancer cause changes in the fluorescence of the mucosa. The disease causes a loss of green fluorescence, making the mucosa appear darker, while normal mucosa usually shows light-green fluorescence [64]; this is due to the disruption in the distribution of those elements that are responsible for autofluorescence in healthy tissues [65] (Figure 8). The advantages of this technique include high sensitivity (estimated 91%) and non-invasiveness, while the main drawback is its low specificity (estimated 58%), since benign conditions such as inflammatory diseases can cause changes in tissue autofluorescence that are not dissimilar to those caused by malignant and pre-malignant conditions [60].

### 3.3. Salivary Biomarkers for Point-of-Care Testing

In the last decade, research has identified biomarkers in biological fluids [66], i.e., saliva, that might have the potential of increasing early diagnosis and detect a premalignant and malignant lesion that is asymptomatic or unnoticeable. Saliva contains many organic and inorganic molecules, proteins, peptides, and electrolytes. Molecular studies have identified more than 100 biomarkers in human saliva [67,68] that acts as indicators of pathological processes and carcinogenesis, such as viruses, cytokines (IL-1b, IL-8, TNF-α), protein receptors (CD44) [68,69,70], and DNA and RNA markers that are overexpressed in a carcinogenic process [68,71]. Since some of these methods are non-invasive, inexpensive, and easy to perform as point-of-care testing technique, they can be well-accepted by patients. However, further research is still required to evaluate and increase the sensitivity and specificity of these testing techniques and if they represent a valid aid for the clinicians to assess the potential and/or presence of malignant transformation of oral mucosa [68,72].

## 4. Conclusions

Early diagnosis of oral cancer is essential to save a patient’s life, minimizing at the same time the negative impact on quality of life that would arise from invasive surgical intervention. Nowadays, there are several diagnostic tools for screening and visual devices that can improve the ability of the clinician to characterize any suspicious lesion, as well as the availability of new point of care testing using salivary biomarkers that recognize the risk of malignant transformation. Although surgical biopsy and histology are still considered the “gold standard”, ancillary methods should be taken into account when performing objective clinical examination. The scientific community is constantly updating preventive measures and screening methods to detect oral cancer at an early stage, intending to reduce the diagnostic delay that in most cases could save the patient’s life.

## Figures and Tables

**Table 1:** Most common clinical signs and symptoms of cancer of the oral mucosa; in the early period the tumor could be totally asymptomatic but evident as a visible aberration of the normal texture and surface of the mucosal lining.

persistent mouth sores and/or pain localized modifications of appearance of the oral mucosa localized modifications of consistence of the oral mucosa persistent white or red or mixed white and red patch of the oral mucosa raised patch or plaque in the oral mucosa persistent lump or growth in the oral mucosa localized bleeding area in the oral mucosa
